# Detrimental Pneumoperitoneum and Bowel Necrosis Secondary to Dilatation and Curettage: Necessitating the Importance of Healthcare Awareness

**DOI:** 10.7759/cureus.18594

**Published:** 2021-10-08

**Authors:** Karanrajsinh Raol, Naveen Kuppusamy, Nairuti A Sanghavi, Navpreet Kaur, Karthikeyan Rajendran

**Affiliations:** 1 Internal Medicine, Gujarat Medical Education and Research Society Medical College and General Hospital, Gandhinagar, IND; 2 Internal Medicine, Government Tiruvannamalai Medical College, Tiruvannamalai, IND; 3 Internal Medicine, Patel, Ramanan & Associates, Waldorf, USA; 4 Internal Medicine, Dayanand Medical College & Hospital, Ludhiana, IND; 5 General Surgery, Government Tiruvannamalai Medical College, Tiruvannamalai, IND

**Keywords:** uterine perforation, spontaneous abortion, small bowel necrosis, pneumoperitoneum, dilation and curettage

## Abstract

We have read about numerous cases depicting life-threatening fatalities due to the lack of awareness and accessibility to health care facilities. A 23-year-old pregnant woman in a village in India underwent a dilation and curettage procedure for spontaneous abortion by an uncertified medical practitioner. Eventually, she presented to the emergency room with ER and an initial abdominal X-ray was consistent with the finding of free air under the diaphragm/pneumoperitoneum and air-fluid levels. Here we present a case of bowel perforation secondary to being strangulated within the uterine cavity.

## Introduction

Dilatation and curettage (D&C) is a common gynecological procedure and is considered to be safe as the mortality ratio associated with it is 0.6 per 1,00,000 legally induced abortions. D&C is indicated for diagnostic and/or therapeutic purposes in either pregnant or non-pregnant patients. When applied to a pregnant patient, it is offered for the first trimester (<14 weeks) elective termination of pregnancy or for management of a missed, incomplete or inevitable abortion [1]. The risk of morbidity and mortality increases with an increase in the age of gestation, parity, and age of the mother. Complications, though rare, can happen and include infection, hemorrhage, cervical lacerations, uterine atony, post-op uterine adhesions (Asherman’s syndrome), and uterine perforations [1,2]. Uterine perforation, the incidence of which is as high as 3.6% in underdeveloped countries, can further lead to bowel/bladder associated complications such as perforation of small bowel/bladder or entrapment of the small bowel, further leading to incarceration, strangulation, and necrosis of the same [3,4]. Female patients with a previous history of cesarean section are at increased risk to develop uterine perforation [3]. Presented here is a case of a 23-year-old female presenting with diffuse abdominal pain, 10 days post a D&C done by medically untrained personnel. It turned out, the procedure had led to perforation of the uterine cavity, further leading to small bowel incarceration, strangulation, and necrosis.

## Case presentation

A 23-year-old Gravida 3, Para 2, Live 2, and Abortion 1 (G3P2L2A1) female presented to the emergency room (ER) with complaints of diffuse lower abdominal pain which had been present for a period of 10 days but was increasingly severe within the past 24 hours. Surprisingly, she denied any nausea, vomiting, abnormal bowel movements, fever, chills, and abnormal vaginal discharge. Vitally, she was tachycardic, tachypneic, and normotensive. It was quite later that her mother mentioned an episode of vaginal bleeding 10 days ago secondary to incomplete abortion in a pregnancy of 11 weeks and a procedure being performed at a remote medical facility that involved ‘cleaning the inside of the uterine cavity' later confirmed to be D&C from the documentation that patient didn’t present in the first place.

On examination, there was diffuse pelvic tenderness with guarding of the abdomen; the patient reported feeling nauseous with one episode of vomiting while in the ER. Apart from the leukocyte count being elevated at 12000/mm3 with a left shift of neutrophils, the routine blood workup was within normal limits. While in the ER, the patient complained of increasing pain and her abdomen was becoming hard to examine due to the guarding. On her vaginal examination, the cervical os was consistent with that of a multigravida with spotting. 

**Figure 1:**
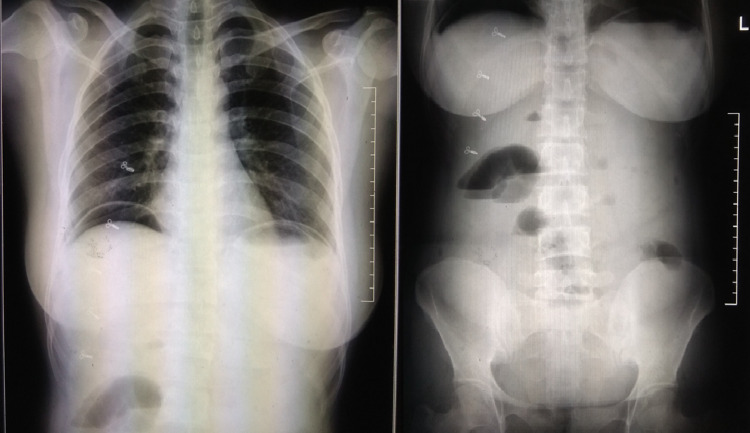
(a [left side]) depicts the free air under both the domes of the diaphragm, (b [right side]) depicts air-fluid levels in the abdomen suggestive of bowel obstruction.

**Figure 2:**
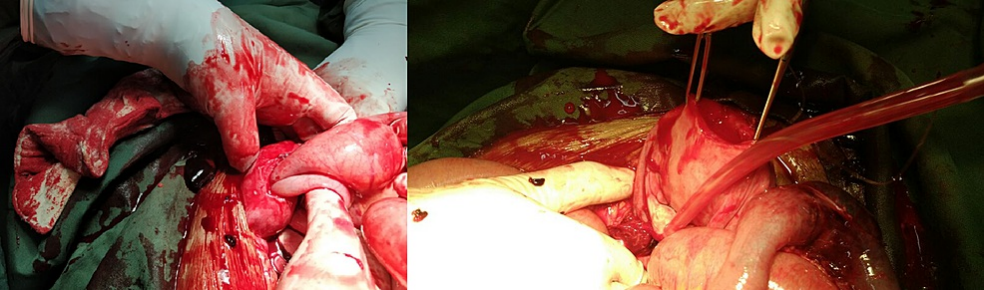
(a [left side]): The strangulated loop of bowel in the uterus through the fundal perforation, (b [right side]): The strangulated bowel loop removed from the perforated uterus.

Orders for abdominal X-ray and pelvic USG were placed; the result of the X-ray was astonishing. It depicted air under both the domes of the diaphragm consistent with pneumoperitoneum (Figure 1a) and multiple air-fluid levels suggestive of bowel obstruction (Figure 1b). Having seen the X-ray, the patient was rushed to the operating room for an emergency laparotomy. Upon opening the abdomen and suctioning 400 ml of blood from the peritoneum, the picture was quite surprising; there was a small bowel loop stuck within the uterus through a massive perforation at the fundus of the uterus measuring approximately 6x3x3 cm (Figure 2a, 2b). The evacuation of the bowel loop with a gentle pull failed. With a multidisciplinary team of surgeons and gynecologists in the operating room, the gynecologist tried reducing the bowel from the uterine cavity per vaginally by dilating the cervix through a Hegar dilator and trying to push the bowel loop into the abdomen, this too in vain.

**Figure 3:**
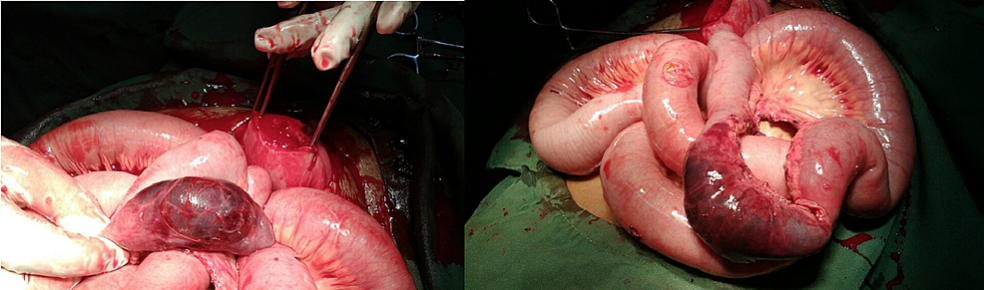
(a [left side]) Gangrenous ileum with a perforated uterus; (b [right side]) 10 cm of bowel necrosis.

After multiple failed efforts, the decision was made to enlarge the perforation of the uterine fundus. Eventually, the strangulated loop of the bowel was able to be evacuated. Upon removal of the bowel loop, 10 cm of the length of the bowel was found to be gangrenous (Figure 3a, 3b). As an identification, it was 50 cm proximal to the ileocaecal junction. The gangrenous portion of the bowel was resected, and the surgeon performed anastomoses of the viable ends. The gynecologist repaired the uterine tear with sutures in layers. Appropriate hemostasis was achieved, two liters of saline wash was done, and the abdomen was closed in layers after placing a pelvic drain. The patient was then shifted to the surgical ICU.

Maintenance fluids were continued throughout the hospital stay. She was nil per oral for 72 hours with a gradual transition to liquid feeds on a postoperative day (POD)-4 to appropriately tolerate solid oral feeds on POD-7. During the postoperative course, prophylactic antibiotics including ceftriaxone, amikacin, and metronidazole were initiated, though being on appropriate antibiotics, she developed spiking fevers on POD-4, complete blood count, blood, urine, and pelvic drain cultures were sent, which did not grow any specific organism apart from the normal flora. Apart from the antibiotics, she received supportive treatments, including GI prophylaxis and IV multivitamins. The patient was shifted to the floors on POD-7, and the pelvic drain was removed on POD-10. She had a good recovery and was discharged on POD-15 with suture removal and an oral antibiotic course, including Cefuroxime, for a period of 14 days.

## Discussion

The patient being in a reproductive age group and considering the history and series of events, the initial set of possible differentials included: endometritis, pelvic inflammatory disease, peritonitis. However, upon the initial X-ray and the laparotomy, the scenario completely changed.

Pneumoperitoneum or free air under the diaphragm can be spontaneous/idiopathic or secondary to hollow viscous perforation. Almost 90% of the cases of pneumoperitoneum have a perforated bowel [5,6]. Certain conditions increase the risk of bowel perforation, including inflammatory bowel disease, infection, diverticulosis, advanced age, constipation, colonic pseudo-obstruction, and pneumatosis cystoides intestinalis [5, 7-12]. In a country like India, bowel perforation could be secondary to infectious aetiologies like typhoid and tuberculosis [13,14]. Gastric perforation could also be secondary to chronic non-steroidal anti-inflammatory drug usage, which leads to a reduction in blood supply to the gastric mucosa and could potentiate the damage to the gastric wall by gastric acid [15].

Apart from the hollow viscus perforation, pneumoperitoneum could be secondary to gynecological factors like vaginal instrumentation and coitus [16]. Corresponding to the timeline of the patient’s presentation was the D&C performed by unauthorized personnel. This could have potentially led to the uterine fundus' perforation due to vigorous scraping while performing curettage. This could be the port of air entry into the peritoneum leading to pneumoperitoneum.

The ileal loop within the uterine cavity could be due to the suctioning after the D&C procedure; this also provided the necessary compression hemostasis to the perforated uterus. In turn, the uterus started contracting back to its normal position creating a ligature mark around the ileal loop, causing an obstruction leading to the presentation of air-fluid levels on the abdominal X-ray. This ligature was enough to cut off the mesenteric blood supply of the ileum and lead to necrosis. Bowel necrosis could have complications like sepsis with systemic hypotension that could lead to end-organ damage, most severely to the kidney and liver [17].

The uterine perforation was the nidus to the patient’s condition. With a history of incomplete spontaneous abortion and lack of awareness mixed with social stigma prevalent in the Indian society revolving around abortion, the patient endangered her life by getting a D&C done at a local uncertified and unauthorized clinic. D&C, though being a simple daycare procedure, could lead to detrimental effects if performed by an unskilled person, as evidenced by our case report.

Having discussed this, the patient at the age of 23 has an extensive uterine fundal scar post the surgery; any future pregnancy would place her at a higher risk of uterine rupture and abnormal placentation, including placenta accreta and in severe cases, it could lead to early miscarriage. Moreover, with an incomplete understanding, she would permanently be traumatized by pregnancy and seek appropriate care for the same past this detrimental incident. She might also develop a sense of mistrust and infidelity towards the whole healthcare system.

This case report depicts the challenges that Indian society faces regarding lack of awareness and access to health care systems. To top it up is the limitation of access to expert medical care facilities. Female reproductive and pregnancy health are the most neglected ones in the rural areas of India, considering the prevailing taboos and the lack of knowledge.

## Conclusions

In today’s era of women empowerment and equality, appropriate awareness and education should be provided to females in rural settings regarding reproductive health, pregnancy, safe abortion practices, and its importance and detrimental effects due to ignorance or inappropriate healthcare practices. This case report depicts the importance of law enforcement against uncertified self-claimed medical practitioners while improving the reach of female reproductive health awareness in remote rural areas to counteract the stigma revolving around the same. Additionally, it also describes the unusual etiology leading to bowel strangulation and necrosis.
